# Spasms and Convulsions

**Published:** 1895-07

**Authors:** A. W. Holcombe

**Affiliations:** Kokomo, Ind.


### SPASMS AND CONVULSIONS.

A. W. HOLCOMBE, M. D., KOKOMO, IND.

*Before the Attack.*

Abdomen bloated. Cup., LACH.
Absent-minded. LACH.
Air, sensation as of, streaming up back into head. ARS.
Angry, for several days. *Bufo.*
Belching. LACH.
Chilliness, then heat. HYOS.
Cloud before left eye, then blindness in left eye. *Tarent.*
Coldness of left side of body. SIL.
Constriction of throat. MOSCH.
Constriction of œsophagus and embarrassed respiration. PLAT
Constriction in pit of stomach. ÆSC-H.
Constriction of throat and chest. CUP.
Contractions, tonic, of muscles. BUFO.
Cough. VERAT-A.
Creeping in the limbs. STRAM.
Creeping sensation from neck down back. LACH.
Chill, intense. *Tarent.*
Drawing in left arm. Cup.
Drawing in limbs. ARS.
Drowsiness. GLON.
Dull feeling in forehead and vertex. GELS.
Dullness and heaviness in head. CUP.
Exaltation of all powers of body. CAN-IND.
Excitable and irritable, for a day. ART-VUL.
Eyes sunken. BUFO.
Face grayish yellow. BUFO.
Face grows red. CUP.
Face pale. LACH., LAUR.
Feet cold. LACH.
Fullness in region of medulla. GELS.
Gaping. Agaric, *Tarent.*

Gasping for breath. LAUR.
Goose-flesh. Cup.
Grasps knees and screams. CIC-V.
Grating teeth. *Sul.*
Headache. IGT., LACH., STRAM.
Hasty drinking. IGT.
Head feels big. GELS.
Head swelled over eyes, suddenly, with horrid pain; sensation as if. *Tuberc.*
Heaviness in head. LACH.
Hungry gnawing. HYOS.
Heat, burning of whole body. ARS.
Intolerance of bed-covers. MOSCH.
Irregular breathing. *Tarent.*
Jaw, lower, dropping of. LAUR.
Jerks in back of neck. BUFO.
Jerking of left arm. CUP.
Lassitude, and tired. GELS.
Labor pains cease and felt in epigastrium. *Ziz.*
Mouse, sensation as if, running through limbs. SIL.
Mouse, sensation as if, running from solar plexus to the brain. SIL.
Mouse, sensation as if, running up arms. SUL.
Mouse, sensation as if, running up back. SUL.
Numbness of left arm and right leg. *Tarent.*
Numbness, feeling of general. BUFO.
Nausea, followed by chill, gaping, and headache. KALI-BI.
Nausea, headache and loss of appetite, for some days. KALI-C.
Nausea and vomiting. *Sul.*
Oppression of chest. MOSCH.
Pain, pressing in fore part of head and over eyes. AST-RUB.
Pains, violent in vertex, some days. *Sul.*
Pains, cutting about heart, and severe chill. PHOS.
Palpitation of heart. Cup., LACH.
Pupils dilated, some days. ARG-NIT.
Respiration, hurried and noisy. CUP.
Restlessness, great. ARG-NIT., MOSCH., ZINC.

Ringing in the ears. Hyos.
Running, sensation as if, something, in the arms. Calc-c.
Running, sensation as if, something, from stomach through abdomen to feet. Calc-c.
Saliva, flow of. Cup.
Screams. Cic-v.
Shaking and twisting of left arm. Sil.
Shrieks, shrill. Cup.
Shuddering, from brain down spine. *Tuberc.*
Sighing. Plb.
Sings and howls out loud. Hyos.
Sleep, deep. *Sul.*
Sparks before the eyes. Hyos.
Speaks unintelligibly. Sil.
Speak, cannot. Cup.
Stopped, sensation as if, everything from right ear to top of head. Phos.
Swallow, desire to, with spasm of pharynx. Calc-c.
Taste, sour, metallic, in mouth. Cup.
Tightness of chest, and acute pains, as if spasm of heart. Hydr-ac.
Trembling. Absinth.
Twitching of whole body for days. Ast-rub.
Twitching of arms and legs, few days. Nat-m.
Unconsciousness. Ars.
Upper lip feels cold and stiff. Lach.
Vertigo. Ars., Hyos., Lach., Plb., Stram., *Sul.*, *Tarent.*
Vertigo, sudden attacks of. Calc-c.
Vomiting of mucus, violent. Cup., Cup-ac.
Vomiting, sudden shriek, then unconsciousness. Hyos.
Vomiting of tasteless water, for some days. Laur.
Weak, becomes so, cannot turn over alone. Sil.
Weeping and laughing alternately. Stram.

*During the Attack.*

Abdomen rises and falls, with rumbling. Ars.
Abdomen puffed up. Ars-s-rub., Stram.

Abdomen sunken. CUP-AC.
Alive, seems half. CROT-HOR.
Abdominal muscles hard as a board. HYDR-AC.
Abdomen bloated. MERC., NUX-V.
Abdominal muscles retracted. TABAC.
Aching in occiput. SEC-C.
Anguish and pressure in chest. HYDR-AC.
Anguish about heart. LYC., *Tarent.*
Arm, rotary motion of left.. *Stram.*
Arms bent at elbow. CIC-V.
Arms thrown from side to side. Cina.
Arms thrown about. Stram.
Arms in constant motion. *Sec-c.*
Arms pressed firmly at sides. HYDR-AC.
Arms jerking. IP.
Bites tongue. Absinth., ART-VUL., BELL., BUFO., *Œnanthe, Op.*
Bites, tongue and cheeks. IGT.
Bandaged, sensation as if the head were, about forehead tightly. INDIG.
Belching, much. IGT.
Body, convulsed. Æth-cyn., AGAR., *Tarent.*
Body, bent back and forward, alternately. BELL.
Body, bent sideways. CHAM.
Body, bluish tint. HYDR-AC.
Body, bent forward. HYDR-AC., NUX-V.
Body, bent backward. Absinth., Acon., Amyg., ARS., BELL., CALC-PH., CHAM., CUP., IGT., LACH., Medorr., *Nux-m.*, NUX-V., OP., STAN., STRAM.
Body, bent backward and suddenly snaps forward. STRAM.
Body, cold. CAMPH., CUP-AC., Œnanthe.
Body, distorted, whole. NAT-M., VERAT-V.
Body, insensible to touch. CAMPH.
Body, motion, in constant. *Sec-c.*
Body, rigid. Acon., CHLORALUM, *Dros.*, IP., Medorr., *Nux-m.*, Œnanthe, SEP., STRAM.
Body, stiff. ÆSC-HYP., Bell., CAMPH., CUP-AC., CUP., MAG-PH., NUX-V.

Body, shaking. *Œnanthe*, VERAT-V.
Body, thrown upward. IGT.
Body, tossed about. MOSCH.
Body, twisted and turned continually. HYOS.
Body, twitches. CHAM.
Body, twitching of, spasmodic. NUX-V.
Body, twitching and trembling. SABAD.
Bloody froth at mouth. IGT.
Breathing hurried and labored. HYDR-AC.
Cheek, one red, and nose cold. IGT.
Choking in throat. CUP-AC.
Closed, left hand, spasmodically. HEPAR.
Clutches at throat. Bell.
Clucking noise as from water poured from bottle, from throat down to abdomen. CINA.
Coldness of thighs. CALC-C.
Conscious. Grat., HELL., HEPAR, IP., MOSCH., NAT-M., NUX-V., PHOS., PLAT., SEP., *Tarent.*
Conscious but can't move or speak. GRAPH.
Constriction, spasmodic, of throat. IGT.
Constriction, spasmodic, of chest. IGT.
Contortion of muscles. CUP.
Convulsed, all over. INDIG.
Cramp in legs. VERAT-V.
Cramp in left hand. HEPAR.
Cramping of chest and sense of suffocation. *Sep.*
Cramps in abdomen and extremities. CUP-AC.
Cries, sharp, from pains in head. ZINC.
Cries when moved. MAG-C.
Cries out. IP.
Cries, shrill. CUP.
Cutting around navel. IP.
Deathly pallor. CHIN.
Distress in epigastrium. *Lyss.*
Double vision. CIC-V.
Drawn up like a ball. CUP.
Draws limbs together. ARS.

Ear, right, cold. Ip.
Emission of semen. Grat.
Elbows drawn behind back and held there. Amyg.
Elbows pressed into sides. *Stram.*
Endeavors to tear everything in reach. Camph.
Eyelids, closed, spasmodically. Hyos.
Eyelids, contracted. Tabac.
Eyelids, paralyzed. Hydr-ac.
Eyelids, paralyzed, upper. Bell.
Eyelids, puffed, upper. Bell.
Eyelids, twitching. Cham., Igt., *Plat.*
Eyes, blue rings around. Cup-ac., Igt., Kali-br., *Stram.*
Eyes bloodshot. Cup-ac.
Eyes constantly moving up and down. *Sul.*
Eyes dim. Cup-ac.
Eyes distorted. Cham., Hyos., Mosch., *Plat.*, Bell., Sil.
Eyes dancing. Phyt.
Eyes drawn to right. Hydr-ac., Ip.
Eyes drawn up spasmodically under lids. Acon.
Eyes fixed. Hydr-ac., *Sul.*, *Tarent.*
Eyes staring. Amyg., Cham., Cup., Hyos., IGT., Kali-br., Ip., Laur., Sil.
Eyes first open, then shut. Ip.
Eyes expressionless. Nux-v.
Eyes open and shut with sudden force. Hyos.
Eyes half open. OP.
Eyes half open and rolling. *Œnanthe.*
Eyes projecting. Hyos., NUX-V.
Eyes open and immovable. Cocc.
Eyes red. Kali-c.
Eyes protruded and glassy. Plat.
Eyes rotated. *Tarent.*
Eyes snapping. Mag-ph.
Eyes squinting. *Tarent.*
Eyes rolling. Cup-ac., Zinc.
Eyes twitching. Stan.
Eyes sunken. Cup-ac.

Eyes turned upward. Bell., GLON., LACH., LAUR., OP., PLAT.
Eyes turned downward. ÆTH-CYN.
Eyeballs twitching. CHAM., IGT.
Extremities cold. CAMPH., *Sul.*
Extremities flexed. BELL.
Extremities jerking. STRAM.
Extremities twitching. CIC-V.
Face, ashy pale. CIC-V.
Face, bloated and dark colored. HYOS.
Face, puffed. OP., STRAM.
Face, swollen. Bell., CROT-HOR., Ip., Œnanthe.
Face, blue. CIC-V., CROT-HOR., CUP., HYDR-AC., HYOS., KALI-BR., *Op.*
Face, cold. CUP-AC., *Nux-v.*
Face, livid. Ast-rub., IGT., Œnanthe.
Face, pale. AST-RUB., CROT-HOR., IP., VERAT-A., ZINC.
Face, pale and sunken. PLAT.
Face, distorted. Absinth., BUFO, CAUST., CHAM., CUP-AC., HYDR-AC., IGT., IP., LAUR., NAT-M.
Face, hot. LAUR.
Face, bluish red. HEP., IP.
Face, red. ÆTH-CY., BELL., BUFO, CUP., GLON., GELS., KALI-C., LAUR., *Stram.*, VERAT-V.
Face, flushed, then pale, then blue. STRAM.
Face, convulsed. ARS., AUR., *Œnanthe.*
Face, sweating. CUP.
Face, right side paralyzed. CAUST.
Face, red and pale, alternately. GLON., KALI-BI., ŒNANTHE.
Face, twitching. IP., LAUR., MOSCH., CHAM.
Features, depressed. CUP-AC.
Feet, cold. CUP-AC.
Feet, drawn up on buttocks. CAUST., CUP.
Feet, cannot be touched on. KALI-C.
Feet, extended. PHYT.
Feet, twitching. STRAM.
Feet, contracted, soles of. VERAT-A.
Fetid breath. KALI-BI.

Fingers, cramps in. VERAT-V.
Fingers, clenched. Mag-ph., MOSCH., *Nux-v.*
Fingers, spasmodically flexed. Kali-c.
Fingers, spread. GLON., Sec-c.
Fingers, spread, of left hand. GLON.
Fever, high. CIC-V.
Fists, clenched across throat. ACON.
Foams at mouth. Absinth., ÆTH-CY., ARS., ART-VUL., AST-RUB., BELL., CEDRON., CHAM., CIC-V., CUP-AC., HYOS., IGT., INDIG., KALI-BI., LACH., LYC., Medorr., NUX-V., OP., *Staph.*, STRAM.
Foams at mouth, large bubbles. HYDR-AC.
Foam, from nose and mouth, bloody. Œnanthe.
Flushes of heat, from abdomen to head. INDIG.
Forearms flexed on arms. HYDR-AC.
Formication and creeping up left arm. HEP.
Gasping for breath. LAUR.
Gnashing of teeth. ACON., CAUST.
Grasps at head and chest. KALI-C.
Grasps and reaches with hands. CHAM.
Grates teeth. Absinth., BUFO, CUP-AC., Fer-mur., HYDR-AC., HYOS., LAUR., *Stram.*, *Tarent.*, *Zinc.*
Grimaces, makes. HYOS.
Grinning features. HYOS.
Groans or sighs occasionally. HYDR-AC.
Groaning. IP.
Hallooing and shouting. CALC-C.
Hands blue. ÆSC-HYP.
Hands and nails blue. NUX-V.
Hands clenched. *Glon.*, LACH., HYDR-AC., PHYT., *Œnanthe*, *Stram.*
Hands clenched, but thumbs not drawn in. LAUR.
Hands cold. CUP-AC., *Nux-v.*
Hands, contortion of, and feet. *Sec-c.*
Hands, alternate contraction of, and feet. Stram.
Hands, automatic motion of, and head. ZINC.
Hands open and shut alternately. Stram.

Hands and feet jerk. CINA.
Hands and limbs jerked upward. *Glon.*
Hand, left, and foot and right eyelid in constant motion. LACH.
Hands thrown above head and become stiff. *Sul.*
Hands and feet tremble. CAMPH.
Hands twitching. STAN., STRAM.
Head drawn back. Amyg., CIC-V., IGT., MAG-C., MOSCH., NUX-V., *Stram.*, TABAC.
Head bent back and sideways. ART-VUL.
Head, constant motion of, and limbs and body. CHLORALUM.
Head, alternately bent back and to side. IGT.
Head, continually thrust to the right, in quick succession. *Stram.*
Head, moved about from side to side. *Sec-c.*
Head, nodded convulsively. NUX-M.
Head, jerked up and down on pillow. STRAM.
Head, jerked from left to right, and from above down. IGT.
Head, rubbed steadily on pillow. HYOS.
Head, rush of blood to. CHIN.
Head, cannot hold up. CUP-AC.
Head, trembling. IGT.
Heart omits every fourth beat. CALC-ARS.
Heart irregular and feeble. HYDR-AC.
Headache, constant. LACH.
Heat in occiput. ZINC.
Hiccoughing. HYDR-AC., IGT.
Inspiration short, expiration long and sighing. IP.
Itching of skin without eruption. *Op.*
Jaws clenched. Acon., ÆTH-CY., Ant-t., Ast-rub., BELL., CEDRON., CUP-AC., HYOS., IGT., MOSCH., *Œnanthe,* PHYT.
Teeth clenched. Fer-mur., GELS., HELL., HYDR-AC., TABAC.
Jaw, under, thrust forward. IGT.
Jactitation of muscles, great. ANT-CRD., ANT-T.
Jerking, of limbs. ART-VUL., *Sep.*
Jerking in inner parts. NUX-M.
Jerking of face. *Sep.*
Jerking, of head, violent. *Sep.*
Kicking with legs. IGT.

Knees drawn up. Amyg.
Laughing and weeping. ALUM., AUR.
Left side of body in constant motion. LACH.
Legs drawn up. *Stram.*
Lies on stomach. CAUST.
Lies on belly and thrusts breech up. CUP.
Limbs, remain in position placed by others. *Stram.*
Limbs, contracted and stretched out slowly. Stram.
Limbs, continually working. *Œnanthe.*
Limbs, drawn up to body and then forcibly thrust out. *Nux-v.*, *Sul.*
Limbs, contracted violently. Fer-mur.
Limbs, alternately contracted and extended. CIC-V.
Limbs, cold. Æth-cy., COFF-CRD., *Nux-v.*
Limbs, convulsed. ARS., AUR., BUFO, CUP-AC., LACH., VER-V.
Limbs, convulsive movements of. Ast-rub., CIC-V.
Limbs, distorted. BELL., Sec-c., Acon.
Limbs, extended. Bell.
Limbs, flexed and rigid. HYOS.
Limbs, stiff. Bell., CUP-AC., HYDR-AC., LAUR., MAG-PH., Medorr., *Millef.*, PHYT., NUX-V.
Limbs, move up and down alternately. CHAM.
Limbs, thrown about. CHAM., CAUST., STAN.
Limbs, trembling. Caust., CROT-HOR., *Sul.*
Limbs, twitching. CHINA, *Dros.*, LACH.
Limb, left, irregular motion of. Cim.
Limb, right, drawn up, left one perfectly straight. MAG-C.
Lips, blue. CUP-AC., *Nux-v.*
Lips, averted and firm. PHYT.
Lips, retracted and showing teeth. HYDR-AC.
Lips, twitching. CHAM., IP., Sil.
Lips and cheeks flabby. NUX-V.
Loquacity and laughing. HYOS.
Loquacity, great. STRAM.
Mental terror. COCC.
Moaning or groaning. *Sil.*

Motions, angular. HYOS.
Motion, muscles all seem to be in. AMYL-N.
Mouth, blue around. *Stram.*, *Sul.*
Mouth, drawn from side to side. CHAM.
Mouth, drawn to one side. CUP-AC.
Mouth, drawn to left side. GLON., HEP., ART-VUL.
Mouth, dry. CUP-AC.
Mouth, discharge of brown mucus from. IP.
Mouth, open, slightly. LAUR.
Mouth, open. MOSCH.
Mouth, jerking sideways and down. MAG-PH.
Muscles, hard as wood. CIC-V.
Muscles, jerking. CROC-SAT.
Muscles, contracted from toes to thighs. Bism.
Muscle, every, in body, in motion. HYOS.
Nausea. CUP-AC., KALI-BR.
Nausea and retching (sometimes). *Stram.*
Neck drawn to right shoulder. CUP.
Neck stiff. CUP-AC., LACH.
Neck and back rigid. TABAC.
Noise in throat as if being choked. *Œnanthe.*
Noises in ears. LAUR.
Nose itching. MERC.
Numbness of legs. PLB.
Pain and rigidity of muscles of back. LACH.
Pain, intense in forehead. Cedron.
Pain in calves. *Nux-v.*
Pain in abdomen and diaphragm. STAN.
Palms of hands contracted. VERAT-A.
Palpitation. CEDRON., *Sec-c.*
Paralysis of one side and convulsions of the other. BELL., STRAM.
Paralysis of muscles of back. CUP-AC.
Pelvis and limbs turned to one side as far as possible. Lyss.
Picking with fingers constantly. ART-VUL.
Pressure in precordial region. CUP-AC.
Pricking and stinging in hands. ÆSC-HYP.

Praying and imploring. STRAM.
Pulse, small, hard, and quick. ÆTH-CY.
Pulse, rapid and weak. Bell.
Pulse, irregular. CEDRON., CUP-AC.
Pulse, quick and small. CUP-AC.
Pulse, feeble. Œnanthe.
Pulse, in long waves. ZINC.
Pulse, wiry. VERAT-V.
Pupils, dilated. ÆTH-CY., Bell., CEDRON., CIC-V., HYDR-AC., IGT., LAUR., OP., *Sec-c.*
Pupils, contracted. PHYT.
Pupils, fixed. ÆTH-CY., Amyg., OP.
Rattling in chest. *Op.*
Resists bending or straightening arms. HYDR-AC.
Respiration, hissing. TABAC.
Respiration, rapid. VERAT-V.
Respiration, imperceptible, almost. *Stram.*
Respiration, labored. *Sec-c.*
Respiration, short and rattling. NUX-V.
Respiration, slow and heavy. *Nux-m.*
Respiration, suspended. LAUR., MOSCH.
Respiration, irregular. IP.
Respiration, interrupted frequently for few moments. CIC-V.
Respiration, rattling. CHAM., HYDR-AC.
Respiration, difficult. CEDRON., *Millef.*, PHYT.
Respiration, stertorous. Bell.
Restlessness, great. CUP-AC.
Rigidity of all muscles. CIC-V.
Rigidity of muscles of neck, limbs, and back. HELL.
Risus sardonicus. Bell., CAUST., CUP-AC., Medorr., Œnanthe.
Roaring in ears. *Plat.*
Rolling of eyes. CHAM., *Sec-c.*
Rolling of head. BELL., POD.
Rolls about and bites at those about him, if disturbed. *Stram.*
Saliva, bloody. BUFO, *Crot-hor.*
Saliva, ropy at mouth. KALI-BICHRO.
Salivation, much. MERC., ŒNANTHE.

Screams, occasional. IGT.
Screaming, loud. KALI-BICHRO.
Shrieks, hoarse. STRAM.
Shrieks. HYOS., LACH.
Shocks in the limbs. Ast-rub.
Shocks that shake whole body. Bar-m.
Skin cool to touch. IGT.
Skin, pale and cold. Ant-t.
Skin, hot. IP., STRAM., ZINC.
Skin, bluish tint. LAUR.
Snapping jerks of lower jaw. BELL.
Spasmodic motion as in coitus. CAUST.
Spasmodic twitching of lower limbs. HELL.
Spitting constantly, but no saliva. HYOS.
Speech impeded or lost. CUP-AC.
Stares straight before her. ART-VUL.
Stares wildly at familiar objects. STRAM.
Stomach distended. CUP.
Stomach and chest blue. Cup.
Sticking in throat, sensation as if something. HEPAR.
Stool, green. HYDR-AC.
Stool, involuntary. CUP., HYDR-AC., ŒNANTHE.
Stool and urine pass involuntary. Art-vul.
Suppression of secretions and excretions. STRAM.
Suffocation. OP.
Suffocation, threatened. HYDR-AC.
Strength of muscles extraordinary. AGAR.
Stretches and writhes. ARS.
Stretching and distortion of head and limbs. Sil.
Sweat, cold, on forehead. VERAT-A.
Sweat, profuse. *Bufo.*, SEP.
Sweat, profuse about head. MOSCH.
Sweat, unpleasant. CUP.
Sweat, violent, offensive. Art-vul.
Talk, incoherent. Kali-c.
Temperature, low. *Chin-sul.* (clinical), CUP-AC.
Tearing hair. *Tuberc.*

Teeth chatter. LAUR.
Throws arms and limbs at right angles with body. OP.
Throws arms about. LYC.
Throws body about. Igt.
Throws himself back. CAMPH.
Thirst, great. CUP-AC.
Tears roll down cheeks. HYDR-AC.
Throat constricted. HYOS.
Thumbs clenched. ÆTH-CY., CHAM., CUP., ŒNANTHE., *Sul.*
Thumbs clenched across palms. GLON., IGT.
Thumbs drawn in. ARUM-TRI.
Thumbs retracted. *Staph.*
Throbbing headache on vertex. HYPER.
Toes spread apart. GLON.
Toes flexed. PHYT.
Tongue swollen. Arum-mac., PLB.
Tongue trembles. CAMPH., IGT.
Tongue paralyzed, right side. CAUST.
Tongue coated and dry. CIC-V.
Tongue partially paralyzed. CUP-AC.
Tongue awry. CUP-AC.
Tongue can't be protruded in straight line. GLON.
Tongue darted in and out like a snake. LACH.
Tongue blue and thick. PLAT.
Tongue hangs from mouth. *Plb.*
Tongue jerked out. *Sec-c.*
Tongue lolling. Sil.
Tongue bitten. *Tarent.*
Tremor, painful. IP.
Trembling in bowels. KALI-BR.
Trembling of whole body, with heat and sweat. LAUR.
Tries to dash head against wall or floor. TUBERC.
Trembling and jerking of limbs. APIS, OP.
Twitching of fingers. CIM.
Twitching of toes. CIM.
Twitching of eyelids and eyeballs. AGAR.
Twitching of cheeks. AGAR.

Twitching in arms. BELL.
Twitching in face. BELL., PLAT.
Twitching of limbs. CHIN-SUL.
Twitching of one side of body. CUP-AC.
Twitching of muscles. DOLICH., HELL.
Twitching of limbs and arms. IGT.
Twitching of corners of mouth. IGT., *Plat.*
Twisting, head. *Kali-bichro.*
Unconscious. Ant-t., ARS., AST-RUB., CIC-V., CROT-HOR., CUP-AC., DOLICH.
Unconscious, without convulsions. CALC-C.
Upper part of body contorted. CIC-V.
Upper and lower extremities alternately convulsed. HYOS.
Urination, involuntary. CUP., HYDR-AC., KALI-BR.

*Attacks with*

Alternation of humor. MOSCH.
Appetite, voracious. Sumb.
Asthma. IPEC.
Body bent back. IPEC.
Breathing, alternation of oppressed. IGT., PLAT.
Consciousness. CINA, KALI-C.
Consciousness, but can't move. Cocc.
Coldness, extreme. HELL.
Cramp, violent, in lower limbs. Cocc.
Cramp, violent, in chest. Cocc.
Cramp, violent, in abdomen. Cocc.
Cries, or involuntary laughter. IGT.
Cries. MERC., *Nux-v.*
Cry, piercing, at each spasm. NUX-V.
Diarrhœa. CALC-PH.
Face, tumid. CEDRON.
Grinding teeth. COFF-CRD.
Hallucinations. KALI-BR.
Heat, feverish, with cold hands and feet. CAUST.
Hiccough. CIC-V., STRAM.
Hilarity. Croc-sat.

Laughter, grimaces and exaltation. CUP-AC.
Laughter, convulsive. PLAT.
Mania. VERAT-V.
Melancholy. INDIG.
Melancholy, and dread of society. CUP-AC.
Micturition, involuntary. CAUST.
Moaning and groaning. LAUR.
Nausea. TABAC.
Nausea, continual. IPEC.
Neck, sense of tight constriction around. CROT-HOR.
Pain, passing down spine to hips. Lyss.
Pain, in stomach. STRAM.
Pain, severe, in back of head. LACH.
Pain, sudden, violent, in abdomen. CHLORALUM.
Rage, alternating with. STRAM.
Screams. Bell., CAUST., CIC-V., CINA, LYC., STRAM.
Sexual intercourse, excessive desire for. CANTH.
Sexual excitement. STRAM.
Shrieks, wild. PLAT.
Sighing and sobbing. IGT.
Spasm of glottis. GELS.
Stools, chalky. CALC-C.
Sweat, cold. TABAC.
Swelling of stomach, as if from spasm of diaphragm. CIC-V.
Stupor. CHAM.
Talking, rapid and confused. MOSCH.
Timidity. INDIG.
Tongue, coated. KALI-BR.
Tongue, coated and dry. CIC-V.
Twitching of limbs, followed by unconsciousness. CUP.
Vomiting. ANT-CRD., CIC-V.
Yawn, frequent inclination to. IGT.

*After the Attack.*

Anxiety in upper abdomen. Ast-rub.
Beautiful, everything seems, even old rags. SUL.
Blind in left eye. *Tarent.*

Breath, cold. Cup.
Breaths, takes deep. LAUR.
Chest, spasmodic constriction of. KALI-C.
Coma, long continued. *Tarent.*
Cough. VERAT-A.
Crawling sensation in arms. MOSCH.
Delirium. *Kali-mur.*
Delirium, active, busy with bed-clothes, resents interference from others. HYDR-AC.
Delirious rage, jumping about and striking those about him. ARG-M.
Diarrhœic stool, one, then constipation. CIC-V.
Disposition changed from irritable to mild and timid. INDIG.
Distress in epigastrium. AST-RUB.
Drowsy, but can't sleep. STRAM.
Dullness of head. CUP.
Eructations. *Chin-ars.*, PULS.
Exhaustion. *Ars.*, ART-VUL., *Chin-ars.*, CUP., HYDR-AC., IGT., *Nat-m.*
Face, deep red and hot. OP.
Faints. Ars-s-fl.
Fever, general. APIS.
Gasping for breath. LAUR.
Hæmoptisis. *Dros.*
Headache. Cup., LAUR., KALI-BR., Kali-c.
Heaviness in head. *Sec-c.*
Hungry, asks for something to eat. *Stram.*
Imagines he is surrounded with friends. HYDR-AC.
Intestines, spasmodic movements of. Bufo.
Lameness. Asc-glab.
Lies on back, draws legs up and spreads them apart. PLAT.
Lies on back for days, unable to speak. *Plat.*
Moans in sleep. *Stram.*
Nausea. PULS.
Numb, left side for two days. ART-VUL.
Oversensitiveness of all the senses. MAG-PH.
Pain and pressure, severe in top of head. BUFO.

Pain, severe in pit of stomach. KALI-BR.
Pain, cramping in region of womb. *Œnanthe.*
Paralysis, apparent. ART-VUL.
Paralysis. CAUST., IP.
Paralysis, symptoms of, remain. *Plb.*
Prostration of limbs, great. *Nux-v.*
Remember, does not, attack. HYOS., IGT.
Respiration, embarrassed and constriction of œsophagus. PLAT.
Screams. Cup.
Shrieks, wild. *Tarent.*
Sighing. IGT.
Sleep, short, soporous. AST-RUB.
Sleep, sound. Bell.
Sleep, profound. BUFO, Canth., HELL., HYOS., IGT., KALI-BR., LACH., NUX-V., *Œnanthe*, OP.
Sleep, snoring. BUFO, *Op.*, PLB.
Sleep. *Dros.*, *Nat-m.*
Sleep, then pain in front of head. *Op.*
Sopor. GLON.
Spots, purple, remain some days. KALI-BR.
Stupid feeling in head. PLB.
Stupor. Absinth.
Sweat, offensive. ART-VUL.
Sweat, cold. *Chin-ars.*
Sweat, general. KALI-C.
Sweat, profuse. IGT.
Sweat, and heat. CALC-C.
Taste in mouth sour. *Sul.*
Talking, laughing, or scolding. HYDR-AC.
Tears, wipes from eyes. SUL.
Tenesmus and strangury, painful. HYOS.
Throbbing, frontal headache. *Tarent.*
Tightness of chest, and acute pains, as if spasm of heart. HYDR-AC.
Tingling in limbs. *Sec-c.*
Tongue, cold. Cup.
Trembles all over. LAUR.

Trembling of right arm. Cup.
Turns and twists till next attack. Cup. (Arg-nit.)
Unconscious. CIC-V., *Œnanthe.*
Unconsciousness lasts. IGT.
Urination of pale urine, profuse. CEDRON., *Cup.*, SUL.
Vertigo. *Tarent.*
Weak. Absinth, AST-RUB., CEDRON.
Weeping. Cup.

*Attack begins with*

Aura from epigastrium. NUX-V.
Blood rushing to head. CALC-ARS.
Coldness, icy from head down back. ARS.
Coldness, over whole body. *Sep.*
Consciousness, loss of. Absinth.
Consciousness, sudden loss of. HYDR-AC., *Tuberc.*
Convulsions, sudden. APIS.
Cries, loud. ŒNANTHE, OP., ZINC.
Cry, a. LACH.
Cry, a wild. BUFO, Cina.
Dizziness. CEDRON., HYDR-AC., INDIG.
Electric shock. ART-VUL.
Falling. Absinth, BUFO, AST-RUB.
Falling, forward. AST-RUB.
Falls, suddenly, with cries and convulsions. HYOS.
Falls, unconscious. LACH., MAG-C., *Sumb.*
Falls, suddenly, with black face. ŒNANTHE.
Falls, suddenly, as if dead, with pale face. *Stram.*
Fidgety feet. ZINC.
Hands, lameness of. KALI-BICHRO.
Headache and tension in spine. NAT-M.
Jerks, sudden, through body. ARS.
Pain in left arm. CALC-ARS.
Pain in heart. CALC-ARS.
Pain, violent, in epigastrium. GLON.
Palpitation, strong. CALC-ARS.
Pressure from epigastrium to throat, thence to head. *Nux-v.*
Rigidity, with sudden jerks. CIC-V.

Rigidity, sudden. MOSCH.
Screaming. CUP-AC., *Stram.*, *Tuberc.*
Screams, followed by faintness or swooning. HYDR-AC.
Shrieks, dreadful. CAMPH.
Shrieking, sudden. KALI-C.
Sigh, long-drawn, and sinks into unconsciousness. BUFO.
Stiffening, sudden, of body. CHAM., CINA.
Twitching of muscles of face about eyes. HYOS.
Twitching in corner of mouth. IGT.
Twitching of hands, then general convulsions. *Sul.*
Vomiting of food. *Hydr-ac.*

*Attack begins in*

Arm. Bell.
Extremities, and spreads over whole body. CUP.
Face. *Dulc.*
Face, twitching, spreads all over body. *Sec-c.*
Fingers and toes. CUP.
Left side and goes to right. *Sul.* (Lach.).
Periphery, and extends upward. CUP-AC.
Plexus, solar. Bufo, INDIG.
Plexus, solar, and spreads to brain. SIL.
Toes. HYDR-AC.
Twitching in hands, then general convulsions. *Sul.*
Upper and lower extremities, and spreads to whole body. *Tarent.*
Wound, a. LEDUM.

*" Part affected."*

Arms. ARUM-TRI., CAMPH., *Can-sat.*, Carb-ac.
Arms and trunk. Can-sat.
Body, one-half, other side lame. APIS.
Body, various parts of, at various times. HYOS.
Back, muscles of. HYDR-AC.
Eyes. Acon.
Feet. Art-vul., CAMPH.
Hands. Art-vul., ARUM-TRI., CAMPH.
Jaws, convulsed, in new-born children. CAMPH.

[TO BE CONTINUED.]

